# Prevalence and Predictors of Soft Drink Consumption among Adolescents in the Gulf Countries: Findings from National Surveys

**DOI:** 10.3390/nu16162637

**Published:** 2024-08-10

**Authors:** Abdulmohsen H. Al-Zalabani

**Affiliations:** Department of Family and Community Medicine, College of Medicine, Taibah University, Madinah 42353, Saudi Arabia; aalzalabani@gmail.com; Tel.: +966-505-654-549

**Keywords:** adolescent, prevalence, carbonated beverages, diet, students

## Abstract

Background and Objectives: This study aimed to investigate the prevalence and predictors of soft drink consumption among adolescents in the Gulf Cooperation Council (GCC) countries using nationally representative data from the Global School-based Student Health Survey (GSHS). Materials and Methods: Cross-sectional data were collected using a self-administered questionnaire in a school survey and included 22,116 adolescents aged 12–18 years from Bahrain, Kuwait, Oman, Qatar, and the United Arab Emirates. Data were collected and analyzed using complex survey methods. The GSHS was not available for Saudi Arabia. Soft drink consumption was the main outcome variable. Multivariable logistic regression was used to examine its associations with sociodemographic characteristics, dietary behaviors, parental factors, and health-related behaviors. Results: The prevalence of soft drink consumption three or more times per day ranged from 10.6% to 26.8% across the countries, with the highest being in Qatar. Adjusted analyses showed that girls had lower odds of SD consumption compared to boys (OR = 0.66; 95% CI: 0.57–0.77). Food insecurity, a proxy for low socioeconomic status, was associated with twice the odds of frequent consumption (OR = 2.06; 95% CI: 1.75–2.43). Parental smoking and low physical activity levels were also associated with higher soft drink intake (OR = 1.46, 95% CI: 1.13–1.88 and OR = 1.18, 95% CI: 1.08–1.28, respectively), while obesity showed a weak positive association. Conclusions: Daily soft drink consumption was highly prevalent among adolescents in the GCC countries. Sociodemographic, behavioral, and health-related factors were significantly associated with frequent intake. These findings highlight the need for comprehensive, multi-sectoral interventions to reduce soft drink consumption in the region.

## 1. Introduction

The global consumption of soft drinks (SD), which are often loaded with high levels of sugar and calories, is escalating, drawing attention from health professionals and policymakers. An international analysis found that the prevalence of consuming one or more SD per day among adolescents ranged from 3.3% to 80% in the 107 included countries [1]. Generally, the consumption of SD globally has shown an increasing trend over the last decade [2,3].

Studies have consistently shown that regular SD consumption is associated with weight gain, obesity, and related health problems in children and adolescents [4,5]. A systematic review and meta-analysis reported that every serving consumed per day can lead to a 0.07 unit increase in the body mass index of children and that consumption reduction interventions were confirmed by randomized controlled trials to prevent weight gain [6]. The health impacts of SD extend beyond weight gain and obesity. Regular SD consumption has been linked to an increased risk of insulin resistance [7], type 2 diabetes [8], asthma [9], and dental caries in children and adolescents [7,10]. De Ruyter et al. found that reducing the consumption of sugar-containing beverages among children significantly reduced weight gain and body fat mass [11]. Another study in Australia found that moving into the top tertile of SD intake was associated with an increase in triglyceride levels and overall cardiometabolic risk among adolescents [12].

These health-related concerns are especially pronounced in younger segments of the population, where the impact of poor dietary choices can be particularly detrimental. Adolescence, a critical period marked by significant physical and cognitive development, also sees the establishment of dietary preferences and habits that are likely to persist into adulthood [7]. Consequently, the nutritional decisions made during this formative phase can set the stage for an individual’s health trajectory well into the future.

The existing literature reveals some factors that could potentially influence the consumption of SD among adolescents. These factors include socioeconomic status, parental influence, the availability and accessibility of SD, as well as the degree and nature of exposure to marketing campaigns [13,14,15,16].

The Gulf Cooperation Council (GCC) countries include Bahrain, Kuwait, Oman, Qatar, Saudi Arabia, and the United Arab Emirates (UAE). The value of addressing SD consumption in the GCC is underscored by evidence of a nutrition transition taking place across the region. This transition is characterized by the increased consumption of processed foods and beverages, coupled with a surge in non-communicable diseases that are often diet related. Factors contributing to this shift include rapid urbanization, robust economic growth, and lifestyle changes that are moving populations away from traditional diets and toward western diets and sedentary routines. Therefore, understanding the patterns of SD consumption among the youth in this region is also important for enriching the global conversation on adolescent nutrition in societies that are experiencing swift modernization. Research on SD consumption among adolescents in the GCC countries is limited. A study found that 56% of adolescents in Jeddah, Saudi Arabia, consumed SD weekly and 17% did so daily [17]. A longitudinal study from Kuwait showed an association between high SD consumption and obesity development [18]. However, gaps remain in the research in the context of the GCC countries. Most existing studies have focused on individual countries, and research on the association between SD consumption and other health behaviors, such as physical activity and dietary habits, in this population is limited.

Therefore, this paper aimed to investigate the prevalence and predictors of consumption of SD among adolescents within the distinct sociocultural environment of the GCC countries using nationally representative data.

## 2. Materials and Methods

### 2.1. Data Source and Sample Characteristics

This study utilized data from the Global School-based Student Health Survey (GSHS), a collaborative surveillance project between the World Health Organization (WHO) and the United States Centers for Disease Control and Prevention (CDC). The GSHS is a nationally representative, cross-sectional survey that collects data on health behaviors and health-related risk factors among adolescents in more than 100 countries worldwide [19].

The current study included the most recent GSHS data conducted in five GCC countries: Bahrain (2016), Kuwait (2015), Oman (2015), Qatar (2011), and the UAE (2016). The discrepancy in the year for Qatar (2011) compared to the other countries (2015–2016) is due to the timing of the most recent GSHS implementation in each country. Saudi Arabia was excluded because GSHS data were not available. In each country, a two-stage cluster sampling design was used to select a representative sample of students aged 12–18 years from public and private schools. The first stage involved selecting schools with a “probability proportional to the size”, while the second stage involved randomly selecting classes within each school. In the selected classes, all students were eligible to participate in the survey.

### 2.2. Variables and Measures

The main outcome variable was SD intake, which was assessed using the question “During the past 30 days, how many times per day did you usually drink carbonated soft drinks, such as Pepsi, Coca-Cola, 7 Up, Sprite, Mountain Dew, Fanta, or any other carbonated soft drinks? (Do not include diet soft drinks.)” Response options included “I did not drink carbonated soft drinks during the past 30 days”, “Less than one time per day”, “1 time per day”, “2 time per day”, “3 time per day”, “4 time per day”, and “5 or more times per day.” For the descriptive analysis in this study, SD consumption was categorized into four levels: “0 times per day” included responses of “I did not drink carbonated soft drinks during the past 30 days”; “less than once per day” included responses of “Less than one time per day”; and “1–2 times per day” and “3 or more times per day” combined corresponding responses. In the multivariable analysis, SD consumption was dichotomized into “0” (none or less than once daily) and “1” (one time per day or more) groups.

The independent variables included sociodemographic characteristics such as sex, age, and food insecurity (which was used as a proxy for socioeconomic status), dietary behaviors (fruit and vegetable intake), parental factors (parental smoking), and health-related behaviors (physical activity and weight status). Food insecurity was measured using the question “During the past 30 days, how often did you go hungry because there was not enough food in your home?” with response options ranging from “never” to “always”. Fruit and vegetable intake was evaluated using one question for each: “During the past 30 days, how many times per day did you usually eat fruit/vegetables?” with response options ranging from “I did not eat fruit/vegetables during the past 30 days” to “five or more times per day”. A combined variable was created by summing the values for fruit and vegetable consumption. A binary variable was then generated to indicate whether participants consumed five or more servings per day of fruits and vegetables (yes/no). Parental smoking was assessed using the question “Which of your parents or guardians use any form of tobacco?” with response options of “neither,” “my father or male guardian,” “my mother or female guardian,” and “both”. Physical activity was assessed using the question “During the past 7 days, on how many days were you physically active for a total of at least 60 min per day?” with response options ranging from 0 to 7 days. The response options were re-coded into a binary variable to indicate whether participants were “physically active for at least 60 min per day on five or more days” (yes/no). Weight status was determined based on self-reported weight and height and then was categorized as “normal weight, overweight, or obesity” using the cutoff values for each age and sex according to the international study by Cole et al. [20] on the standard definition of overweight and obesity.

### 2.3. Statistical Analyses

All analyses were conducted using Stata version 18.0 (“StataCorp LLC, College Station, TX, USA”). The complex survey design was accounted for using the “svy” command, with sampling weights, clustering, and stratification variables provided by the GSHS. Descriptive statistics were used to summarize the characteristics of the study population and the prevalence of SD consumption by country and sex. Associations between SD consumption and the independent variables were examined using logistic regression models, with “none or less than one time per day” as the baseline group. The models were adjusted for age, sex, country, and other covariates in the model (food insecurity, fruit and vegetable consumption, parental smoking, physical activity, and weight status). Separate models were run for the overall sample and by sex. Odds ratios (ORs) with 95% confidence intervals (CIs) were reported.

### 2.4. Ethical Approval

This study analyzed existing, de-identified public-use data from the GYTS, which were approved by ethics research review boards for each country.

## 3. Results

### 3.1. Sample Characteristics

The sample sizes ranged from 2021 in Qatar to 7141 in Bahrain. The final sample included 22,116 adolescents aged 12–18 years from five GCC countries: Bahrain (32.3%), Kuwait (16.4%), Oman (15.7%), Qatar (9.1%), and the UAE (26.4%). Table 1 presents the sample characteristics by country. Overall, 57% of participants were aged 13–15 years and 51.4% were female. Only 23% of participants consumed fruits and vegetables five or more times per day. Parental smoking was reported by 20.5% of adolescents, with 2.8% stating that both parents smoked. About one-quarter (24.9%) were physically active, and 24.4% had obesity based on self-reported weight and height.

### 3.2. Prevalence of SD Consumption

Table 2 shows the prevalence of SD consumption by sex and country. Overall, 10.6% to 26.8% of adolescents consumed SD three or more times per day, with the highest prevalence observed in Qatar (26.8%; 95% CI: 23.9–30.0%). The proportion of adolescents who did not consume any SD was highest in Bahrain (22.0%; 95% CI: 20.0–24.3%) and lowest in Qatar (15.1%; 95% CI: 12.6–18.0%). SD consumption three or more times per day was more prevalent in boys than in girls in all GCC countries except Qatar.

### 3.3. Factors Associated with SD Consumption

The multivariable logistic regression results of variables associated with SD consumption at least once daily are presented in Table 3. In the overall sample, the odds of consuming SD were higher among adolescents aged 13–15 years (OR = 1.20; 95% CI: 1.01–1.43) and 16–18 years (OR = 1.24; 95% CI: 1.01–1.52) compared to those aged 12 years or younger. Girls had lower odds of SD consumption compared to boys (OR = 0.66; 95% CI: 0.57–0.77). Food insecurity (a proxy for socioeconomic status) was associated with higher odds of SD consumption, with adolescents experiencing food insecurity most of the time or always having twice the odds of frequent consumption compared to those who were never food insecure (OR = 2.06; 95% CI: 1.75–2.43). Surprisingly, adolescents who consumed fruits and vegetables five or more times per day had slightly higher odds of frequent SD consumption (OR = 1.14; 95% CI: 1.04–1.25), although in stratified analysis, this held true for girls only. Parental smoking was associated with higher odds of SD consumption, particularly when both parents smoked (OR = 1.46; 95% CI: 1.13–1.88). Physically active adolescents had lower odds of frequent SD consumption (OR = 0.85; 95% CI: 0.78–0.93). Obesity was associated with slightly higher odds of frequent consumption compared to normal weight, although this association was not statistically significant.

## 4. Discussion

This study examined the prevalence and correlates of SD consumption among adolescents in five GCC countries using nationally representative data from the GSHS. The findings showed a high prevalence of daily SD consumption across all countries, with significant variations by sex and country. The study also identified several sociodemographic, behavioral, and health-related risk factors associated with SD consumption, including sex, age, food insecurity, fruit and vegetable intake, parental smoking, physical activity, and weight status.

The prevalence of daily SD intake observed in this study is at the higher end of the spectrum across other parts of the world (ranging from 3.3% to 80% in 107 countries) [1]. Alhareky et al. reported a similarly high prevalence of consumption of SD in Kuwait (57.9% moderate and 7.7% high soda consumption) [18]. However, the results of the present study contrast starkly with the prevalence of SD consumption in European countries, where the average was reported to be 16% [21]. The sex differences in SD consumption observed in this study, with boys being more likely to consume SD daily than girls, have also been reported in previous studies from the GCC region [22] and other countries [23,24,25].

The high prevalence of soft drink consumption observed in this study aligns with global high prevalence, especially in countries undergoing rapid urbanization and economic growth [2]. This high level is particularly concerning given the well-established link between sugar-sweetened beverage consumption and various health issues. Long-term consequences may include increased rates of obesity, type 2 diabetes, and dental caries among youth. A systematic review by Malik et al. [5] found strong evidence for the association between SD consumption and weight gain in children and adolescents. Furthermore, habitual consumption of SD was associated with a greater incidence of type 2 diabetes, independent of adiposity [7,8].

The association between low socioeconomic status (as proxied by food insecurity) and SD consumption found in this study is consistent with research from other countries showing that adolescents from low-income households are more likely to consume sugary drinks than those from high-income households [26,27]. This may be due to the lower cost and greater availability of sugary drinks compared to healthier beverages in low-income settings, as well as the use of sugary drinks as a coping strategy for stress and anxiety associated with food insecurity [28]. Therefore, healthy eating interventions need to account for social, structural, and organizational factors operating in socioeconomically disadvantaged populations [29,30].

The positive association between fruit and vegetable intake and SD consumption observed in this study is somewhat surprising given that previous research has shown an inverse association between these behaviors [31,32]. However, other studies have shown mixed results. A study of secondary school students in Australia showed an association between SD consumption and fruit but not vegetable intake [24]. One possible explanation is that adolescents who consume more fruits and vegetables may also have greater access to and availability of SD at home and school, leading to higher consumption of both types of food.

The inverse association between physical activity and SD consumption observed in this study is also consistent with previous research [22] and may reflect the displacement of physical activity by sedentary behaviors such as television viewing and video gaming, which are often accompanied by the consumption of sugary drinks [33].

Finally, the association between weight status and SD consumption found in this study, particularly among girls, is consistent with the well-established link between sugary drinks and obesity in children and adolescents [4,6]. This finding highlights the need for interventions and policies that target the reduction of sugary drink consumption as a strategy for obesity prevention and control in the GCC countries.

Given the high prevalence of SD consumption and its association with various health risks, it is necessary to implement effective interventions at various levels (national, school, healthcare, and family) to reduce SD intake among adolescents. At the country level, implementing sugar-sweetened beverage taxes has been shown to reduce sugary drink consumption in various populations, including adolescents [34,35]. These taxes can be combined with public awareness campaigns that highlight the health risks associated with excessive sugary drink consumption and promote healthier beverage choices. School-based interventions have shown promise in promoting healthy eating behaviors and reducing sugary drink consumption [36]. These interventions may include nutrition education programs and policies that restrict the availability and sale of SD on school premises while ensuring access to safe drinking water and healthy beverage options [37,38]. A randomized controlled intervention trial in five countries in the region (including Saudi Arabia and Bahrain from the GCC) showed a school-based program to be effective in reducing the consumption and purchase of SD [39]. Healthcare providers can focus on lifestyle changes when counseling adolescents [40,41] using lifestyle medicine tools such as motivational interviewing [42]. At the family level, interventions such as family-based education programs and home-based interventions can help create supportive environments that encourage healthy eating behaviors and limit the availability of SD at home [43].

The strengths of this study include the use of nationally representative data from multiple GCC countries, the large sample size, and the examination of multiple sociodemographic, behavioral, and health-related correlates of SD consumption. However, the study also has some limitations. First, the cross-sectional design of the GSHS precludes the inference of causal associations between the examined variables. Longitudinal studies are needed to confirm the directionality of these associations and to identify causal determinants of soft drink consumption. Second, the self-reported data used may be subject to recall and social desirability biases, particularly for health-related behaviors such as SD consumption and weight status. The use of self-reported weight and height may have led to misclassification of weight status, potentially affecting the observed associations between weight status and soft drink consumption. Third, the GSHS questionnaire assessed only the frequency of soft drink consumption without information on portion sizes. This lack of data on portion sizes may have led to an incomplete picture of overall dietary intake. Fourth, some subgroups had relatively small sample sizes, which may have affected the precision of our estimates and the power to detect significant associations. Finally, the study did not examine the influence of other important factors such as school environment, food marketing, and public policies on SD consumption among adolescents in the GCC countries.

## 5. Conclusions

This study found a high prevalence among adolescents of daily SD consumption in five GCC countries, with significant variations by sex and country. These findings emphasize the need for comprehensive and multi-sectoral interventions that address the various determinants of SD consumption among adolescents in the GCC countries. Such interventions may include school-based programs to promote physical activity and a healthy diet, policies that restrict the availability and marketing of sugary drinks, and public awareness campaigns that educate adolescents and their families about the health risks of excessive sugary drink consumption. Additional research is needed to evaluate the effectiveness of these interventions in reducing SD intake and improving the health outcomes of adolescents in the GCC countries.

## Figures and Tables

**Table 1:** Characteristics of participants by country (unweighted number and percentages).

	Bahrain	Kuwait	Oman	Qatar	UAE	Total
Sample (unweighted)	7141 (32.3%)	3637 (16.4%)	3468 (15.7%)	2021 (9.1%)	5849 (26.4%)	22,116 (100.0%)
Age						
12 or younger	1204 (16.9%)	85 (2.4%)	79 (2.3%)	517 (25.9%)	322 (5.5%)	2207 (10.0%)
13–15	4339 (60.8%)	1990 (55.2%)	1630 (47.2%)	1414 (70.7%)	3190 (54.8%)	12,563 (57.0%)
16–18	1594 (22.3%)	1530 (42.4%)	1747 (50.5%)	69 (3.5%)	2314 (39.7%)	7254 (32.9%)
Sex						
Male	3685 (51.7%)	1657 (46.5%)	1623 (47.7%)	912 (45.9%)	2763 (47.6%)	10,640 (48.6%)
Female	3449 (48.3%)	1906 (53.5%)	1781 (52.3%)	1075 (54.1%)	3041 (52.4%)	11,252 (51.4%)
Food insecurity						
Never	3220 (45.2%)	1840 (51.0%)	2130 (61.8%)	1347 (69.5%)	2979 (51.2%)	11,516 (52.5%)
Rarely/sometimes	3238 (45.5%)	1491 (41.3%)	1149 (33.4%)	449 (23.2%)	2257 (38.8%)	8584 (39.1%)
Most of the time/always	666 (9.3%)	277 (7.7%)	166 (4.8%)	143 (7.4%)	581 (10.0%)	1833 (8.4%)
Fruit and Vegetables (5 or more per day)						
No	5486 (76.8%)	2985 (82.1%)	2658 (76.6%)	1389 (68.7%)	4509 (77.1%)	17,027 (77.0%)
Yes	1655 (23.2%)	652 (17.9%)	810 (23.4%)	632 (31.3%)	1340 (22.9%)	5089 (23.0%)
Parental smoking						
None	5820 (82.2%)	2191 (64.6%)	3077 (89.9%)	1399 (76.1%)	4590 (79.9%)	17,077 (79.5%)
One parent	1092 (15.4%)	1071 (31.6%)	310 (9.1%)	347 (18.9%)	980 (17.1%)	3800 (17.7%)
Both parents	170 (2.4%)	129 (3.8%)	35 (1.0%)	92 (5.0%)	176 (3.1%)	602 (2.8%)
Physically active						
No	4878 (68.5%)	2511 (75.2%)	2881 (84.6%)	1591 (84.7%)	4275 (74.5%)	16,136 (75.1%)
Yes	2239 (31.5%)	830 (24.8%)	526 (15.4%)	287 (15.3%)	1460 (25.5%)	5342 (24.9%)
Weight status						
Normal	4310 (60.4%)	1778 (48.9%)	2322 (67.0%)	517 (25.6%)	3405 (58.2%)	12,332 (55.8%)
Overweight	1607 (22.5%)	819 (22.5%)	535 (15.4%)	233 (11.5%)	1194 (20.4%)	4388 (19.8%)
Obesity	1224 (17.1%)	1040 (28.6%)	611 (17.6%)	1271 (62.9%)	1250 (21.4%)	5396 (24.4%)

**Table 2:** Soft drinks consumption by adolescents in GCC countries by sex and country (weighted proportion and 95% confidence intervals).

	Bahrain	Kuwait	Oman	Qatar	UAE
Overall					
0 time per day	22.03 (19.96–24.26)	17.06 (14.07–20.52)	20.24 (18.03–22.65)	15.11 (12.62–17.99)	20.06 (17.33–23.09)
<1 time per day	43.37 (40.81–45.97)	28.65 (25.84–31.64)	36.22 (33.19–39.37)	24.56 (22.26–27.01)	45.54 (42.72–48.38)
1–2 times per day	23.65 (21.69–25.73)	37.52 (34.13–41.04)	30.27 (27.15–33.59)	33.52 (30.79–36.35)	23.77 (20.8–27.03)
≥3 times per day	10.94 (9.13–13.07)	16.77 (13.94–20.04)	13.27 (11.53–15.22)	26.82 (23.9–29.95)	10.63 (9.03–12.48)
Boys					
0 time per day	20.35 (18.57–22.25)	14.67 (11.35–18.77)	17.36 (14.78–20.28)	17.52 (13.15–22.98)	16.07 (14.13–18.21)
<1 time per day	39.52 (37.04–42.05)	29.03 (24.12–34.5)	32.96 (29.44–36.69)	25.91 (22.25–29.95)	43.85 (40.71–47.05)
1–2 times per day	26.46 (24.45–28.58)	38.96 (34.41–43.71)	34.1 (30.56–37.82)	31.34 (27.71–35.21)	26.31 (23.75–29.05)
≥3 times per day	13.67 (11.29–16.47)	17.33 (13.17–22.47)	15.58 (12.82–18.8)	25.22 (20.94–30.05)	13.76 (11.32–16.64)
Girls					
0 time per day	23.78 (20.7–27.16)	19.3 (15.81–23.36)	22.84 (19.95–26.01)	12.57 (9.97–15.74)	24 (19.68–28.93)
<1 time per day	47.4 (44.95–49.85)	28.53 (25.29–32.01)	39.81 (36.75–42.94)	23.72 (20.88–26.83)	47.35 (44.13–50.6)
1–2 times per day	20.7 (18.48–23.12)	36.25 (32.14–40.58)	26.27 (23.31–29.47)	35.41 (32.05–38.92)	21.23 (17.48–25.53)
≥3 times per day	8.12 (6.7–9.81)	15.91 (12.87–19.52)	11.08 (9.1–13.43)	28.3 (24.31–32.66)	7.42 (5.93–9.24)

**Table 3:** Association of soft drinks consumption and various risk factors in the overall sample and stratified by sex.

	Overall Adjusted OR (95% CI) *	Boys Adjusted OR (95% CI)	Girls Adjusted OR (95% CI)
Age			
12 years or younger	Ref.	Ref.	Ref.
13–15 years	1.2 (1.01–1.43)	1.28 (0.98–1.66)	1.15 (0.93–1.43)
16–18 years	1.24 (1.01–1.52)	1.4 (1.04–1.9)	1.11 (0.87–1.41)
Sex			
Male	Ref.	-	-
Female	0.66 (0.57–0.77)	-	-
Food insecurity			
Never	Ref.	Ref.	Ref.
Rarely/sometimes	1.22 (1.1–1.34)	1.26 (1.11–1.41)	1.18 (1.03–1.35)
Most of the time/always	2.06 (1.75–2.43)	1.89 (1.51–2.37)	2.23 (1.84–2.7)
Fruit/vegetables >5/d			
No	Ref.	Ref.	Ref.
Yes	1.14 (1.04–1.25)	1.13 (0.99–1.29)	1.16 (1.01–1.33)
Parents smoke			
No	Ref.	Ref.	Ref.
One parent	1.31 (1.17–1.47)	1.26 (1.1–1.43)	1.38 (1.15–1.65)
Both parents	1.46 (1.13–1.88)	1.3 (0.96–1.75)	1.64 (1.15–2.34)
Physically active			
No	Ref.	Ref.	Ref.
Yes	0.85 (0.78–0.93)	0.86 (0.77–0.97)	0.83 (0.72–0.96)
Weight status			
Normal	Ref.	Ref.	Ref.
Overweight	0.89 (0.81–0.98)	0.85 (0.74–0.96)	0.94 (0.84–1.06)
Obesity	1.09 (0.99–1.2)	1.04 (0.9–1.19)	1.14 (1.01–1.29)

* All models were adjusted for all variables in the table in addition to country.

## Data Availability

The data presented in this study are available in the WHO “NCD Microdata Repository” at https://extranet.who.int/ncdsmicrodata/index.php/home (accessed on 16 July 2024). These data are available in the public domain.
